# SARS-CoV-2 outbreaks on Danish mink farms and mitigating public health interventions

**DOI:** 10.1093/eurpub/ckab182

**Published:** 2021-10-08

**Authors:** Torben Dall Schmidt, Timo Mitze

**Affiliations:** 1 Institute of Employment Relations and Labour (IPA), Helmut Schmidt University, Hamburg, Germany; 2 Department of Business and Economics, University of Southern Denmark, Odense, Denmark

## Abstract

**Background:**

First severe acute respiratory syndrome coronavirus 2 (SARS-CoV-2) infections on Danish mink farms were reported in June 2020 and thereupon spread geographically. We provide population-level evidence on excess human incidence rates in Danish municipalities affected by disease outbreaks on mink farms and evaluate the effectiveness of two non-pharmaceutical interventions, i.e. culling of infected mink and local lockdowns.

**Methods:**

We use information on SARS-CoV-2 outbreaks on mink farms in 94 Danish municipalities together with data on human SARS-CoV-2 cases and tested persons in Weeks 24–51 of 2020. Difference-in-difference estimation and panel event studies for weekly human incidence rates are applied to (i) identify epidemiological trends of human SARS-CoV-2 infections associated with disease outbreaks on mink farms, and (ii) quantify the mitigating effects from the two non-pharmaceutical interventions.

**Results:**

SARS-CoV-2 outbreaks on mink farms in a municipality associate with an increase in weekly human incidence rates by about 75%; spatial spillover effects to neighbouring municipalities are also observed. Local lockdowns reduce human incidence rates, while culling of mink appears to be more effective in combination with a lockdown. The temporal lag between an outbreak on a mink farm and a significant increase in human incidence rates is estimated to be 1–3 weeks; lockdowns and culling of mink neutralize this effect 4–8 weeks after the initial outbreak.

**Conclusions:**

SARS-CoV-2 infections among farmed mink in Denmark significantly link to local human infection trends. Strict animal and human disease surveillance in regions with mink farming should be pursued internationally to mitigate future epidemic developments.

## Introduction

The rapid global spread of the severe acute respiratory syndrome coronavirus 2 (SARS-CoV-2) and high infection rates among Danish farmed mink^1^ (Neovison vison) emphasize the importance of surveilling epidemiological trends for human population associated with infected farmed mink. We complement previous virological research that studies the link between infected mink and human disease cases.^2–6^ Our main objective is to assess whether municipalities with infected farmed mink bear the risk of becoming hot spots of human SARS-CoV-2 infections from underlying two-way infection dynamics between animals and humans. We focus on infection effects from such two-way dynamics and not on specific causal relationships through, e.g. zoonotic transmission.

Two observations may render these two-way dynamics stronger in certain regions. First, the share of SARS-CoV-2 infected humans among households living at Danish mink farms reached an average level of 19% between June and November 2020 and was even higher in the north-western part of the country (30%).^7^ These higher shares point to a systematic link between mink farm employment and SARS-CoV-2 infections. Second, the industry structure is geographically concentrated with 3529 out of all 3942 registered jobs at 1248 mink farms in 2018 being located in the western part of the country.^8^ As persons living and/or working at mink farms with a high infection rate interact with the surrounding society, this may increase population-level infection rates in those municipalities affected by a local SARS-CoV-2 outbreak on mink farms in an accumulative manner. This is what we analyze in the following.

From a public health perspective, different non-pharmaceutical interventions including travel restrictions, physical distancing, face masks, hand hygiene and phases of lockdowns have been imposed to curb SARS-CoV-2 infections among humans.^9–12^ We focus on two non-pharmaceutical interventions, which were introduced in Denmark to suppress the spread of human SARS-CoV-2 infections in municipalities affected by SARS-CoV-2 outbreaks on mink farms, i.e. culling of mink and a local lockdown in seven severely affected municipalities. Other measures, such as wearing of face masks, hand hygiene, social distance rules and restrictions on entering mink farms, were also in place during our sample period. However, these measures were applied to all mink farms across Denmark, why our estimation approach implicitly controls for this when we include municipal fixed effects.

We compare human incidence rates in Danish municipalities before and after a first disease outbreak on a mink farm in a municipality with and without policy interventions in place. As infections may spread beyond municipal borders, we also consider effects on neighbouring municipalities. Our focus is on studying the epidemiological link between animal-to-human and human-to-human viral transmission. Large reservoirs of SARS-COV-like viruses in horseshoe bats have been a persistent concern.^13^ SARS-CoV-2 in farmed minks has occurred in eight countries worldwide including Denmark by 3 December 2020.^14^ The Netherlands, Spain and Poland have like Denmark introduced local culling of infected mink. Insights into infection trends associated with mink and the role of mitigation measures of SARS-CoV-like viruses is accordingly of international relevance.

## Methods

### Study population and period

For our 2020 data, a total of 1147 mink farms were located in 61 of the 98 Danish municipalities. SARS-CoV-2 outbreaks on at least one mink farm were reported in 24 municipalities. This offers the opportunity to compare SARS-CoV-2 infections in human populations living in municipalities with SARS-CoV-2 outbreaks on mink farms to those without as an increasing number of municipalities experienced SARS-CoV-2 outbreaks on mink farms from June 2020 until end-December 2020. Testing and reporting of infections in mink relied on standardized procedures of the Danish Veterinary and Food Administration. Legislation obliged mink farmers to report suspicions of infections, which arguably reduces risks of identification bias from hidden infections in farmed mink lending some credence to our method.

**Figure 1:**
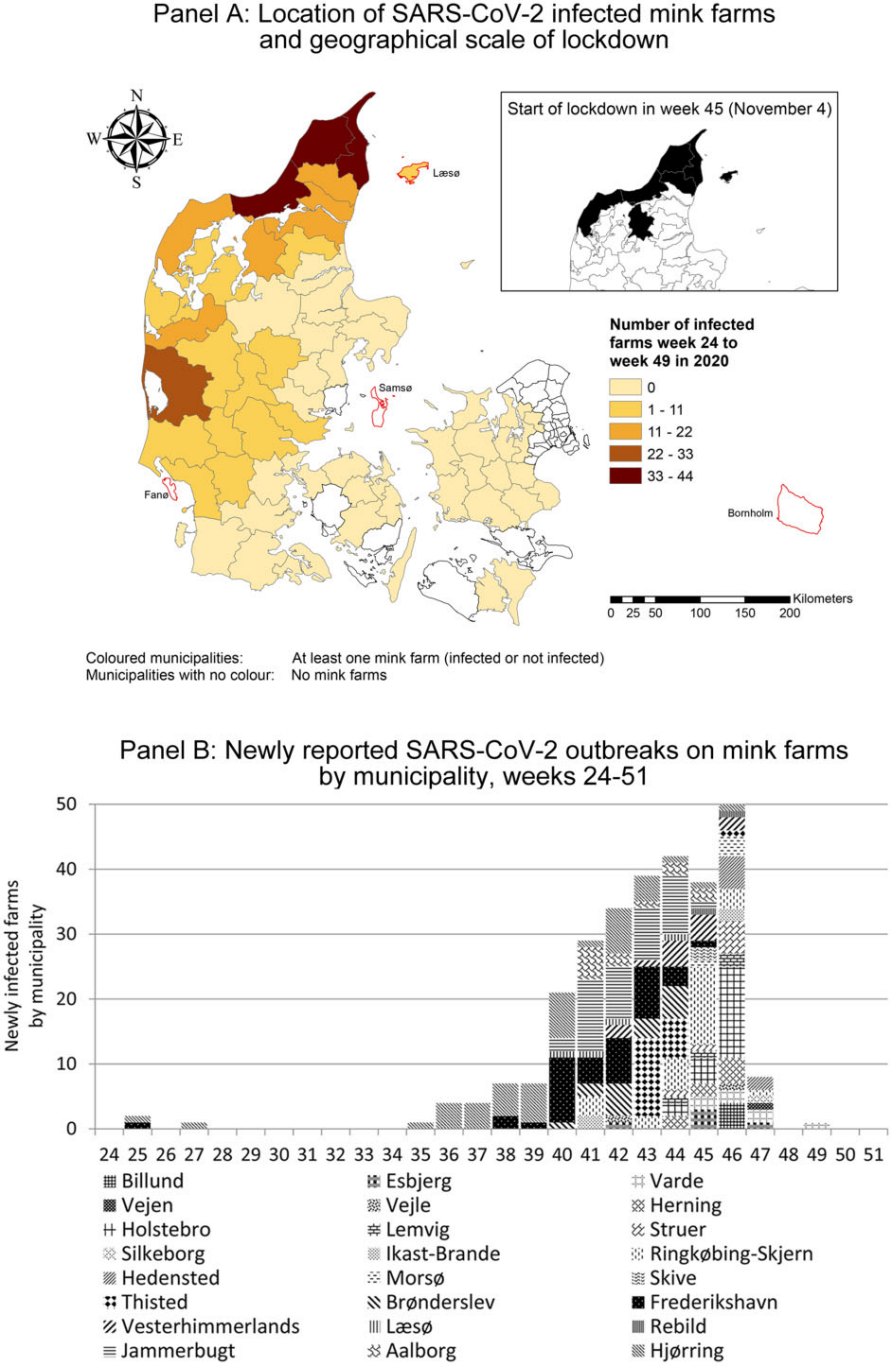
Lockdown, infected mink farms and non-infected mink farms. Notes: Supplementary appendix figure A2 additionally presents the cumulative stocks calculated on the basis of data on new outbreaks on mink farms. The municipalities with a red border are the excluded 4 island municipalities of Fanø, Læsø, Samsø and Bornholm. Source: Fødevarestyrelsen, Smittede mink farme uge for uge (in Danish, foedevarestyrelsen.dk). Retrieved: 8 April 2021.

The geographical distribution of infected and non-infected mink farms is shown in figure 1A, while figure 1B shows the weekly number of newly infected mink farms in different municipalities. First infected mink farms were reported in two municipalities in the north-western part of Denmark in June 2020 (calendar Weeks 24–26). The number of infected farms increased rapidly and spread geographically from mid-September (Week 38). The presence of reservoir hosts of SARS-CoV-2 in farmed mink therefore increased from Weeks 24–49. A number of non-pharmaceutical interventions were implemented. We focus on culling all farmed mink, which was largely accomplished by end of 2020, and a local lockdown in seven severely affected municipalities between Week 45 and 49 (see figure 1A or Supplementary appendix box A1 and figure A1). Our study population is accordingly SARS-CoV-2 infected human residents in Danish municipalities and our study period spans the period from first infected mink farms in Week 24 until Week 51 in 2020.

### Data sources

Daily data on new SARS-CoV-2 infection cases and number of tested persons in the human population for each of the 98 Danish municipalities are retrieved from the official COVID-19 Dashboard governed by Statens Serum Institut.^15^ Published data on weekly outbreaks of SARS-CoV-2 on mink farms in Danish municipalities are obtained from the homepage of the Danish Veterinary and Food Administration.^16^ Process data on infection and culling of mink is furthermore obtained from the Danish Veterinary and Food Administration. We provide details in the Supplementary appendix on how we combine these data for robustness tests. Data on workplace mobility are taken from the Google COVID-19 Community Mobility Report informing on mobility to workplace locations (categorized by Google) in municipalities. Values are deviations from a normal inflow defined as the median inflow from 3 January 2020 to 6 February 2020.^17^ Climate information by weather station in terms of average weekly temperature is retrieved from the metObs data of the Danish Meteorological Institute applying an API procedure.^18^ Each municipality is assigned the temperature value of the weather station that is geographically closest to the municipality’s centroid.

Data have been retrieved on 8 April 2021 and cover calendar Weeks 24–51 in 2020 for 94 Danish municipalities (2632 observations). Of the total of 98 municipalities, we leave out 4 island municipalities for which data are incomplete. All data are aggregated by week and municipality to match the frequency of data on infected mink farms. We also include information on the type of municipality by socio-economic structure.^19^ Social structures have previously been shown to be important for disease transmission and COVID-19 fatalities.^20^ Summary statistics can be found in the Supplementary appendix tables A1–A3.

The key outcome variable is the weekly incidence rate per municipality, which counts the number of new human SARS-CoV-2 infections per 100 000 human inhabitants of a municipality by calendar week. Testing is based on PCR tests. To investigate the robustness of results, the positivity rate is an alternative outcome variable, i.e. the percentage share of individuals tested positive for SARS-CoV-2 in all tested persons per municipality and calendar week.

### Statistical analysis

The staggered occurrence of SARS-CoV-2 infections in farmed mink offers the possibility to test for the link between these outbreaks and SARS-CoV-2 human incidence rates. The lockdown on November 4 in seven municipalities combined with culling of infected mink furthermore presents an opportunity to test for the effectiveness of each of these mitigation measures by public health authorities.

We use static and dynamic treatment estimation models to investigate how SARS-CoV-2 outbreaks on mink farms relate to human SARS-CoV-2 infections in municipalities. Our baseline utilizes a static treatment approach based on a difference-in-difference (DiD) model, which is a widely applied tool for inference in public health policy research, in general, and SARS-CoV-2, in particular.^21–24^ We compare human SARS-CoV-2 infections across municipalities with and without infected mink farms (first difference) before and after first infections on mink farms in a municipality (second difference). Figure 1A presents municipalities with and without infected mink farms.

Our baseline is an absorbing treatment indicator, which takes values of one from the week onwards for which the first infection on a mink farm in a given municipality is reported; it is zero before that week. This absorbing specification assumes that SARS-CoV-2 outbreaks on mink farms have a potentially permanent effect on the local human population in a municipality after the treatment starts and for the remainder of the sample period. As a sensitivity check, we also define non-absorbing binary treatment indicators, i.e. binary dummies that take values of one only in the first 3–4 weeks after the first infection on a mink farm in a municipality and revert back to zero afterwards, unless a new outbreak on another farm in the same municipality extends the treatment period subsequently. The use of a 3–4 weeks effect window is motivated by a median human incubation time of ∼1–2 weeks and equally lengthened infection duration. Through these non-absorbing binary treatment indicators, we can investigate if potential excess infection trends associated with SARS-CoV-2 outbreaks on mink farms are of transitory nature.

The assumption of (permanent or transitory) time constant, static treatment effects in the DiD framework may be overly restrictive. We thus also compute dynamic treatment effects making use of panel event studies (PES). This framework allows for a fully flexible time heterogeneity in the link between SARS-CoV-2 outbreaks on mink farms and population-level human incidence rates.^25–28^ Estimated parameters are allowed to vary by week without making assumptions on the duration of effects unlike in the DiD framework – though at the cost of only measuring net effects from infected mink farms, culling and lockdowns. The PES estimates indicate a positive peak in effects about 3–4 weeks after first infections on mink farms. This supports our choice of a time window of 3–4 weeks for our non-absorbing binary treatment in the DiD framework. Another type of sensitivity check considers continuous (rather than binary) treatment variables in the DiD framework that accumulate reported mink farm (or animal) infections in each municipality, either as permanent stocks or on a rolling basis over 3–4 weeks. Formal presentations of the DiD and PES estimation setup are given in the Supplementary appendix.

Mitigating effects from two different non-pharmaceutical interventions are considered in the DiD framework. A lockdown in seven municipalities severely affected by SARS-CoV-2 infected mink was implemented by the Danish government, which is measured by a binary indicator for the seven municipalities taking values of one from calendar Week 45 onwards (see figure 1A). Culling of mink on farms ordered by the Danish government is considered by including the number of infected mink farms culled in different municipalities by week. To account for transmission lags between culling and human incidence rates, we accumulate culling of mink livestock on infected mink farms over a 3 weeks period.

We use data from all 98 Danish municipalities excluding the 4 island municipalities of Fanø, Læsø, Samsø and Bornholm. Additionally, we also restrict the analysis to a subsample of 60 municipalities comprising municipalities with at least one mink farm, excluding the island municipality of Læsø (see figure 1A). This subsample may closer align underlying characteristics of treated and comparison municipalities. Given municipalities with more infections on mink farms may see stronger testing effort for humans that would confound our results, we control for the number of human PCR tests conducted in a municipality in the current and last two weeks. Colder weather and workplace mobility may mirror human behaviour that leads to an increase in human SARS-CoV-2 infections irrespective of mink infections, why we include average temperature and workplace mobility (lagged by 1 week to account for a time delay in the reporting of new SARS-CoV-2 infections).^29^ Other latent confounding factors are controlled for by including municipality- and week-specific indicators. Finally, we control for linear and quadratic time trends for four different region types capturing social and economic structures (urban, intermediate urban, rural and periphery) that may influence human SARS-CoV-2 infections irrespective of outbreaks on mink farms.

Next to the municipal’s own average number of human infections (over the last 2 weeks), we also account for the number of human infections in neighbouring municipalities (over the last two weeks) as a channel for human-to-human disease transmission that may confound our treatment effect.^30^ In similar veins, we include variables to capture spatial spillovers from culling of infected mink and local lockdowns. We classify any two municipalities as neighbours if their centroids fall within a 50-km distance radius and define binary neighbourhood dummies that take values of one if a disease outbreak (or a lockdown or culling infected mink) takes place in a neighbouring municipality. We exclude directly treated municipalities in the calculation of neighbourhood dummies to avoid a double counting. For continuous variables, neighbourhood effects are measured as the average variable value across neighbouring municipalities.

## Results

For our baseline specification with absorbing treatment shown in Column (1) of table 1, the estimated link between SARS-CoV-2 outbreaks on mink farms and weekly human incidence rate in the same municipality is an increase by 51.25 cases per 100 000 human population (95% CI: 30.910, 71.591). Given an average weekly human incidence rate of 68.26 (see Supplementary appendix table A2) in comparison municipalities during the treatment period, this treatment effect translates into a 75% increase in weekly human incidence rates. Neighbouring municipalities to those affected by local disease outbreaks on mink farms also experience a rise in the incidence rate, although the spatial indirect effect is smaller than the direct effect.

**Table 1:** DiD estimation results for human SARS-CoV-2 incidence rate

Sample	(1) All municipalities	(2) All municipalities	(3) All municipalities	(4) Only municipalities with mink farms
Infected farms	51.25			28.21
(absorbing binary)	(30.910, 71.591)			(6.226, 50.199)
Infected farms^a^		74.03		
(non-absorbing, binary)		(48.432, 99.633)		
Infected farms^a^			6.22	
(continuous)			(4.174, 8.264)	
Neighbour (50 km)	21.79	69.02	7.33	−9.96
Infected farms	(5.470, 38.099)	(31.588, 106.461)	(1.835, 12.818)	(−40.216, 20.297)
Lockdown Northern	−46.01	−44.19	−29.94	−52.60
Jutland (binary)	(−84.731, −7.279)	(−78.956, −9.432)	(−60.268, 0.391)	(−88.894, −16.307)
Neighbour (50 km)	−22.06	−21.61	−7.47	−89.71
Lockdown Northern Jutland	(−52.288, 8.164)	(−52.451, 9.241)	(−36.281, 21.343)	(−171.09, −8.333)
Culling on infected	−21.67	−29.13	0.58	−5.16
mink farms (binary)	(−42.153, −1.180)	(−55.276, −2.982)	(−17.123, 18.288)	(−27.513, 17.201)
Neighbour (50 km)	−14.09	−13.54	4.84	0.12
Culling on infected mink farms	(−29.907, 1.725)	(−29.671, 2.588)	(−11.093, 20.765)	(−14.080, 14.324)
Number of infectious human	0.07	0.06	0.06	0.13
population (continuous)	(0.028, 0.112)	(0.026, 0.101)	(0.027, 0.093)	(0.067, 0.202)
Neighbour (50 km)	0.20	0.14	0.18	0.05
Number of infectious human population	(0.118, 0.278)	(0.061, 0.225)	(0.096, 0.254)	(−0.021, 0.117)
Cumulative number of	−0.001	−0.001	−0.001	−0.002
PCR tests (continuous)	(−0.0021, −0.0004)	(−0.0019, −0.0004)	(−0.0016, −0.0003)	(−0.0029, −0.0010)
Workplace mobility	0.26	0.47	0.36	−0.20
(continuous)	(−0.135, 0.658)	(0.089, 0.846)	(−0.025, 0.739)	(−0.627, 0.222)
Average temperature	−9.51	−6.07	−6.76	−7.81
(continuous)	(−14.777, −4.249)	(−11.174, −0.962)	(−11.975, −1.535)	(−11.864, −3.765)
No. of observations	2632	2632	2632	1680
No. of clusters (municipalities)	94	94	94	60
Within *R*^2^	0.78	0.78	0.78	0.74
Linear and quadratic time trends by region types	Yes	Yes	Yes	Yes
Week and municipal fixed effects	Yes	Yes	Yes	Yes

Notes: 95% confidence intervals in parenthesis.

a Variables defined on a 3-week rolling basis (see main text and Supplementary appendix for details on variable definitions); neighbour values for the variable ‘Infected farms’ are measured in the same dimension as the underlying variable in each column.

Effects become generally larger if we move to a non-absorbing (transitory) binary indicator that reverts back to zero after 3 weeks for a treated municipality [see Column (2) of table 1]. Using a continuous variable that accumulates the number of infected farms based on a 3-week rolling time window points to an increase in the weekly human incidence rate of 6.22 cases per 100 000 population (95% CI: 4.174, 8.264) for each additional SARS-CoV-2 outbreak on a mink farm [see Column (3) of table 1]. Given the average weekly value of 10.7 for the number of infected farms for a 3-week rolling time window, this translates into an average increase in the incidence rate by 66.5 cases per 100 000 human populations. Hence, the results in Columns (2) and (3) indicate that the rise in incidence rates is particularly strong in the first 3 weeks after the disease outbreak. If we reduce the sample size to only those 60 municipalities with mink farms, we find qualitatively similar though smaller treatment effects [see Column (4) of table 1].

With regard to the effects of the two non-pharmaceutical interventions included in this study, for the period of the lockdown in the seven municipalities, the weekly human incidence rate decreases by between −29.94 (95% CI: −60.268, 0.391) and -52.60 (95% CI: −88.894, −16.307) cases per 100 000 human population per week. Over the ∼3 weeks of the lockdown, the cumulative reduction is at 90–156 cases per 100 000 human populations. With a human population in the seven municipalities of ∼280 000 humans, a simple back of the envelope calculation shows that this translates into an estimated reduction of 252–436 human cases associated with the lockdown. In our baseline specification in Column (1) of table 1, the reduction in human incidence rates from culling of infected mink is at −21.67 cases per 100 000 human populations (95% CI: −42.153, −1.180).

**Figure 2:**
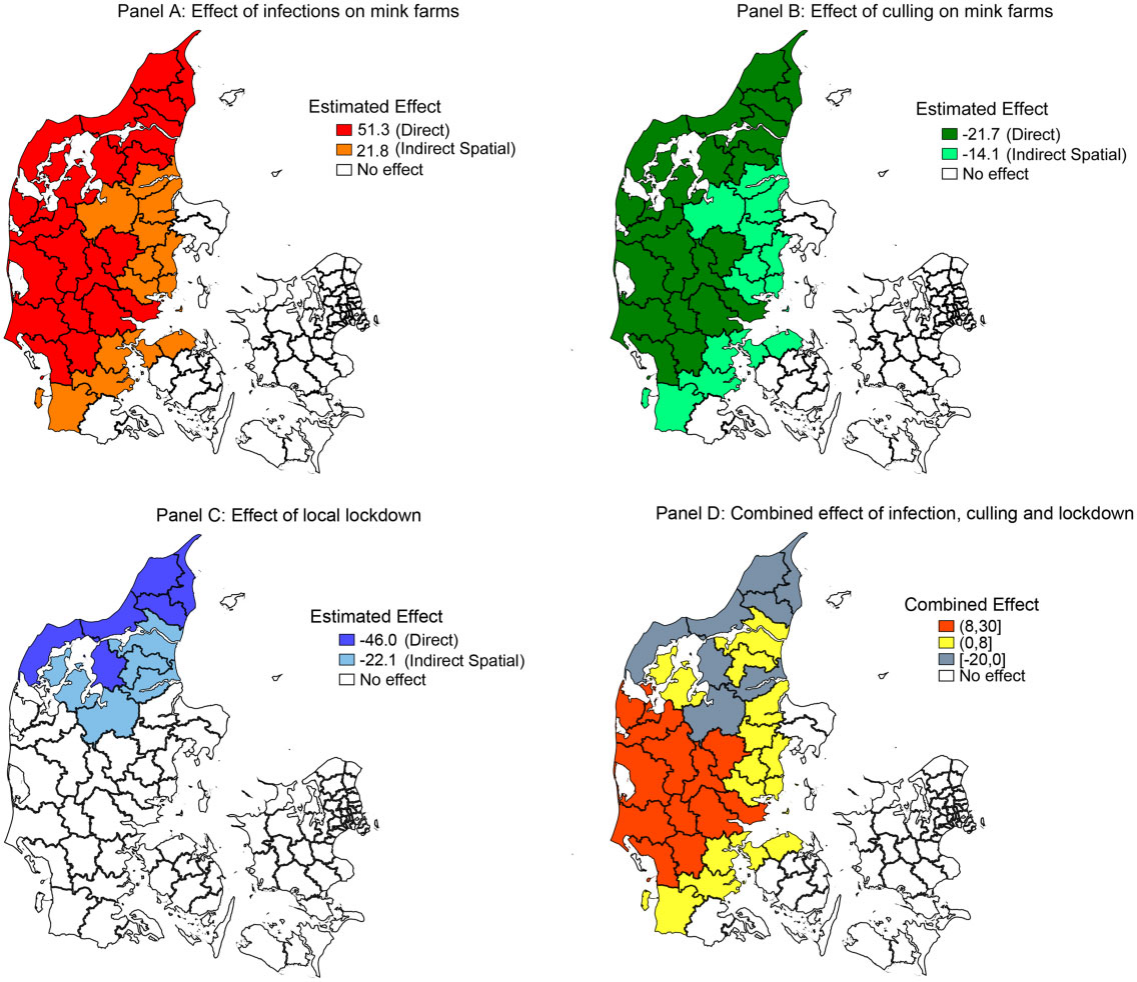
Partial and combined effects on human SARS-CoV-2 incidence rate from infected mink farms and public health interventions. Notes: Panel (D) summarizes the combined effects of Panels (A), (B) and (C) for the period Week 24 until Week 51 in 2020. Estimated parameters in Column (1) in table 1 are combined for each municipality considering binary for SARS-CoV-2 infections on mink farms, binary for culling on infected mink farms, binary for lockdown and binaries for neighbours to municipalities experiencing these events within a 50 km from the centroid. The inclusion of spatial dependence results in the exclusion of four island municipalities, specifically Fanø, Læsø, Samsø and Bornholm. Particularly, Læsø was among 61 municipalities with infected mink farms. Source: Based on difference-in-difference approach using method and data described in section on data and statistical analysis.

Figure 2 presents the spatial distribution of estimated direct (same municipality), spatial indirect (neighbouring municipalities) effects of infections on mink farms in a municipality and the working of the two non-pharmaceutical interventions for the baseline model in Column (1) of table 1. Figure 2D combines partial effects estimated by our statistical model from the treatment effect from SARS-CoV-2 outbreaks on mink farms [Panel (A)], culling [Panel (B)] and lockdown measures [Panel (C)]. The combined reduction of human SARS-CoV-2 incidence rates to levels before any SARS-CoV-2 outbreak on mink farms are only achieved in certain municipalities, namely those with a lockdown and their neighbours. This protective effect is indicated by the grey areas in figure 2D.

**Figure 3:**
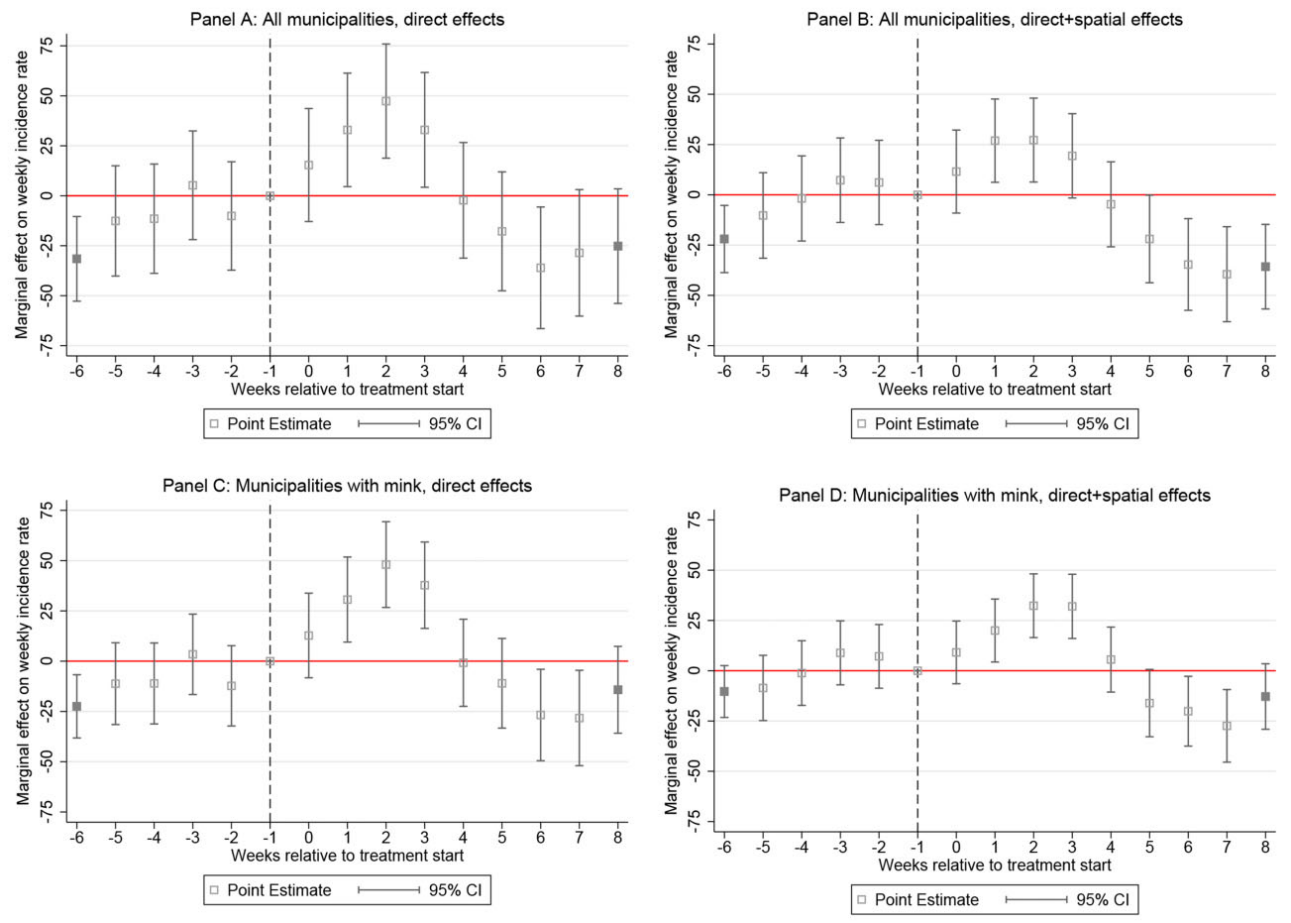
PES on the effect of infected mink farms in municipalities. Notes: Panels (A) and (B) for all 94 municipalities, Panels (C) and (D) for 60 municipalities with at least one mink farm. Source: Based on panel event studies using method and data described in section on data and statistical analysis.

As the results in table 1 have already pointed at time-heterogeneous effects comparing absorbing and non-absorbing treatments, figure 3 reports the fully flexible PES estimation coefficients for weekly treatment effects on the human incidence rates in treated municipalities over a maximum of 6 weeks before and 8 weeks after the first SARS-CoV-2 outbreak on a mink farm in the municipality. Estimated effects beyond this time interval are accumulated in a single coefficient shown in the first pre- and last post-treatment period. The last pre-treatment observation, i.e. the week before the first disease outbreak, is used as baseline period and no effect is estimated for this week.

Importantly, the plotted coefficients should be interpreted as combined effects for municipalities of SARS-CoV-2 outbreaks on mink farms, culling of mink and a local lockdown as we only control for the timing of treatment start (outbreak) but not the type of treatment (policy interventions). In figure 3, we report time-heterogeneous effects for directly treated municipalities and spatial indirectly treated municipalities. For the latter group, statistically significant positive effects are in Panels (B) and (D) observed about 1–3 weeks after the first SARS-CoV-2 outbreak on a mink farm. Thereafter, the combined effect of culling mink and the local lockdown appears to reduce incidence rates consecutively. Specifically, in weeks 4–5 after treatment start the mitigating interventions already neutralize effects on human incidence rates and in weeks 6–7 incidence rates fall significantly below the reference period. Considering results with only direct effects in Panels (A) and (C) of figure 3 sees increases in week 1–3 after the first SARS-CoV-2 outbreaks on mink farms and reductions in weeks 6–7, which are declining towards week 8. Sensitivity checks for different outcome variables and alternatively specified models are shown in Supplementary appendix tables A4–A6 and figure A3.

## Discussion

Outbreaks of SARS-CoV-2 animal infections on mink farms first occurred in June 2020 in a few municipalities in the north-western part of Denmark. The number of infected mink farms increased from late August 2020 and spread geographically from mid-September 2020 constituting reservoir hosts for increased animal and human SARS-CoV-2 infections. The Danish government responded by first culling on infected mink farms and adjacent mink farms in a radius of 7.8 km but eventually all mink in Denmark. It also implemented a 3-weeks local lockdown in the most severely affected municipalities on 4 November 2020. This study contributes to the nascent empirical literature on the link between SARS-CoV-2 infections in mink and human incidence rates and evaluates the effectiveness of two non-pharmaceutical interventions.

We find evidence of up to 75% higher human incidences rates in Danish municipalities with SARS-CoV-2 outbreaks on mink farms compared to municipalities with no outbreaks of SARS-CoV-2 on mink farms. Human disease effects arrive 1–3 weeks after the occurrence of the first disease outbreak on a mink farm in a municipality and its neighbouring municipalities. From a public health perspective, it is notable that human SARS-CoV-2 incidence rates are reduced with the lockdowns and culling of mink, which could indicate that these mitigating measures are able to curb spread of infections. Containment effects become effective between 4 and 8 weeks after the occurrence of the first SARS-CoV-2 outbreak on a mink farm in a municipality. The results indicate an effect of lockdowns, while the combined effect of SARS-CoV-2 infections on mink farms, culling and lockdowns is only negative for locked down municipalities and their neighbours. This may point in the direction that lockdowns and culling of mink should go hand in hand. Using our estimated model parameters, we find that the local lockdowns were able to reduce the number of human infections by up to 436 over a 3-week period. This estimate is at the lower end of computer simulations based on a SEIHR epidemiological model reporting a lockdown-induced reduction of 500–800 cases.^31^ However, we in addition find significant spatial indirect effects, which have to be added for a full assessment of local lockdown. The seven municipalities with a lockdown and their neighbouring municipalities were also in addition affected by culling of mink.

SARS-CoV-2 outbreaks on mink farms have been reported in a number of countries (Lithuania, the Netherlands, Spain, Sweden, Italy, Greece and USA). Our findings may therefore be helpful for effective SARS-CoV-2 surveillance and public health strategies in a number of countries. While uncertainties associated with the data (e.g. process data) and assumptions underlying the statistical model may to some extent prevail in the analysis, our empirical approach has carefully assessed the robustness of results to such assumptions and data structures.

Disease transmission among both humans and animals is a concern.^32^ Previous examples of respiratory transmission are SARS in 2003,^33^ N1H1 virus in 2009^34^ and MERS-COV in 2012.^35^ We do not claim that our results can be extrapolated exactly into these other examples, but rather that the emergence of disease with respiratory transmission which may have a two-way infection dynamic among humans and animals is recurrent in history. Our results provide evidence on the importance of surveillance of human and animal infections in such situations and on possible combinations of public health measures that are effective to curb the viral spread.

## Supplementary data

Supplementary data are available at *EURPUB* online.

## Supplementary Material

ckab182_Supplementary_Data
