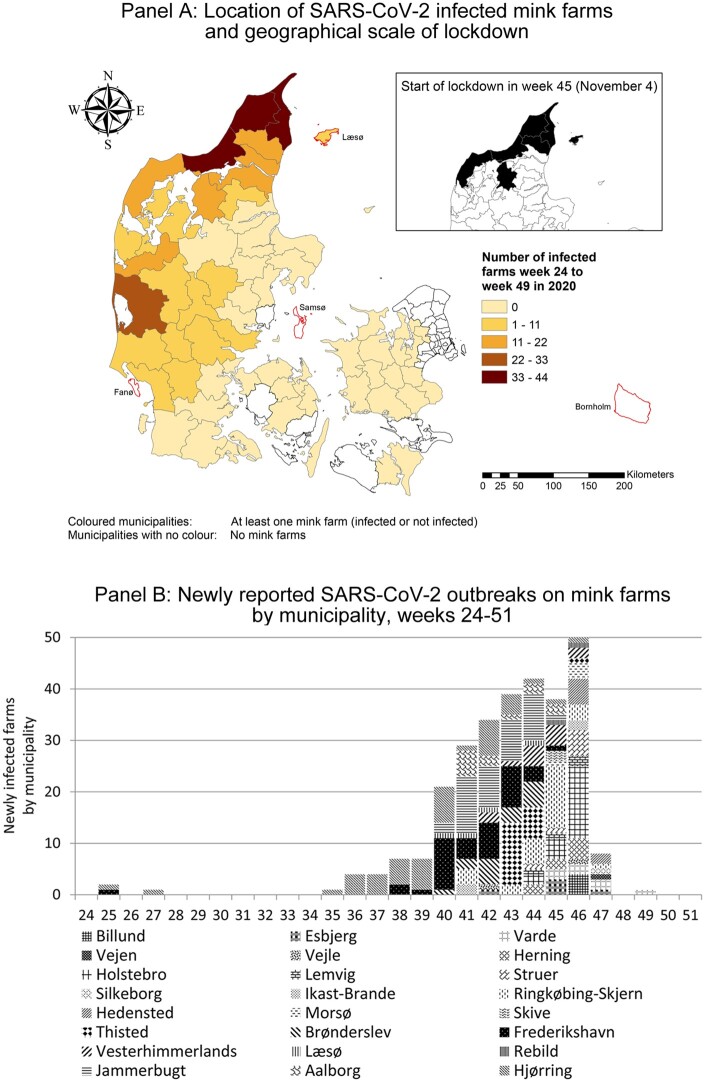# Corrigendum to: SARS-CoV-2 outbreaks on Danish mink farms and mitigating public health interventions

**DOI:** 10.1093/eurpub/ckab215

**Published:** 2022-01-12

*European Journal of Public Health*, 2021, https://doi.org/10.1093/eurpub/ckab182

In the originally version of the article, Figure 1 was published:


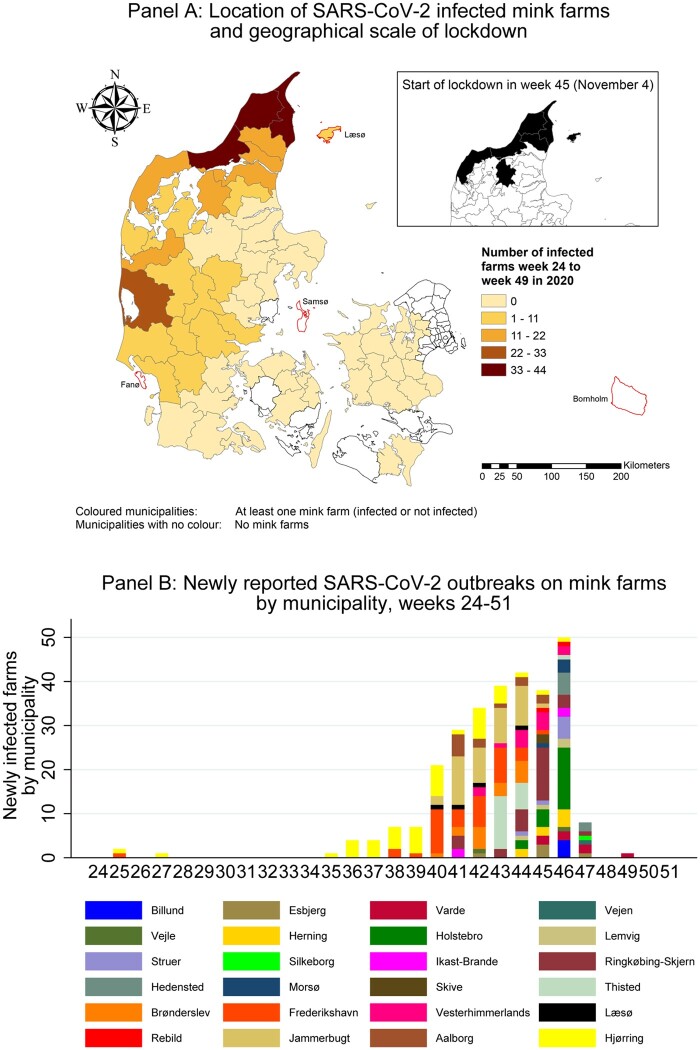


but is now revised as follows: