# Supplementary material for: SARS-CoV-2 outbreaks on Danish mink farms and mitigating public health interventions
Source: Eur J Public Health. 2021 Oct 8;32(1):151–7. doi: 10.1093/eurpub/ckab182 (PMC8549281; doi:10.1093/eurpub/ckab182)
Supplement: ckab182_Supplementary_Data [file ckab182_supplementary_data.pdf]

## SI Appendix

## "SARS-CoV-2 outbreaks on Danish mink farms and mitigating public health interventions"

**Torben Dall Schmidt and Timo Mitze**

### A. Extended method description

***Difference-in-Difference estimation (DiD).*** Our default DiD model specification employs a spatially augmented two-way fixed effects (FE) framework specified as[1-2]

$$Y_{i,t} = \delta_1 \cdot InfFarm_{i,t} + \delta_2 \cdot WInfFarm_{i,t} + \rho_1 \cdot Lockdown_{i,t} + \rho_2 \cdot WLockdown_{i,t} + \theta_1 \cdot Culling_{i,t}$$

$$+ \theta_2 \cdot WCulling_{i,t} + \beta_1 \cdot Cum\_Infectious_{i,t} + \beta_2 \cdot WCum\_Infectious_{i,t} + \tau \cdot Cum\_Tested_{i,t}$$

$$+ \vartheta \cdot L.Temperature_{i,t} + \lambda \cdot L.Mobility_{i,t} + \sum_{r=1}^{R} \gamma_r \cdot \left(RegType_{r(i)} \times Trend_t\right)$$

$$+ \sum_{r=1}^{R} \alpha_r \cdot \left(RegType_{r(i)} \times Trend_t^2\right) + \tau_t + \mu_i + \varepsilon_{i,t}, \qquad (A.1)$$

where $Y_{i,t}$ denotes the human SARS-CoV-2 incidence rates for municipality $i$ in calendar week $t$ and $InfFarm_{i,t}$ is the treatment indicator for SARS-CoV-2 infection outbreaks on Danish mink farms. In the baseline specification, this variable is an absorbing treatment indicator, which takes values of one for municipality $i$ from week $t$ onwards for which the first infection on a mink farm in this municipality is reported; it is zero before that date and for municipalities not affected by a disease outbreak in week $t$. The parameter $\delta_1$ tests for the link from SARS-CoV-2 infections on mink farms to SARS-CoV-2 infections in the human population of municipality $i$; the parameter $\delta_2$ similarly tests for the link between SARS-CoV-2 infections on mink farms and SARS-CoV-2 infections in the human population of neighbouring municipalities to municipality $i$. The underlying spatial treatment indicator $WInfFarm_{i,t}$ takes values of one, if a municipality $j \neq i$ is located in the spatial neighbourhood of municipality $i$ experiencing a disease outbreak on a mink farm. Two municipalities $i$ and $j$ are classified as geographical neighbours if the centroids of these municipalities lie within a 50km radius. From $WInfFarm_{i,t}$ we exclude all municipalities that are included in $InfFarm_{i,t}$ to avoid a double counting of treatment effects.

Two factors of mitigating measures (non-pharmaceutical interventions) in public health policy are included. First, the lockdown in seven severely affected municipalities in Northern Jutland is measured by a binary dummy variable. The variable $Lockdown_{i,t}$ accounts for the potential mitigating effect of the local lockdown in seven municipalities in Northern Jutland from calendar week 45 (i.e., it takes values of one for seven municipalities during the weeks 45 to 49 and is zero otherwise, see Panel A in Figure 1 in the main manuscript for a visualization of locked down municipalities). The local lockdown was motivated by the ambition to curb human SARS-CoV-2 infections in municipalities particularly affected by SARS-CoV-2 infections in farmed mink, why the parameter $\rho_1$ tests for the effectiveness of this mitigating measure. The variable $WLockdown_{i,t}$ covers geographical neighbours of lockdown municipalities (again, based on a 50 km radius) and the coefficient $\rho_2$ accordingly tests for the presence of spatial spillovers for this mitigating measure.

Second, a political decision to cull mink on infected mink farms and at later stages all mink farms was decided by the Danish Government as a further mitigating measure next to the local lockdowns. The variable $Culling_{i,t}$ is a binary dummy that indicates whether a farm with infected mink has been subject to culling in a given municipality *i* and week *t*. To account for transmission lags from culling of mink to human incidence rates, we accumulate the number of culled mink on infected mink farms over a period of three weeks for the definition of $Culling_{i,t}$. To capture potential spatial spillover effects from culling, $WCulling_{i,t}$ measures whether a municipality is a neighbor to a municipality subject to culling ($WCulling_{i,t} = 1$) or not. The parameters $\theta_1$ and $\theta_2$ therefore tests for the effectiveness of a public health policy stressing culling of mink to curb human SARS-CoV-2 infections, directly or through spatial spillovers.

The estimations additionally account for the stock of human infections in a municipality and its spatial neighbourhood term ($Cum\_Infectious_{i,t}, WCum\_Infectious_{i,t}$) in order to control for human-to-human SARS-CoV-2 transmission dynamics. The variable $Cum\_Infectious_{i,t}$ accumulates the number of SARS-CoV-2 cases in a municipality over the last two weeks and $WCum\_Infectious_{i,t}$ measures the average number of reported human infections in neighbouring municipalities (based on a 50km radius) over the same period. Further, we control for the number of PCR tested persons per municipality in the current and

previous two weeks ($Cum\_Tested_{i,t}$). The motivation here is that the local implementation of testing strategies may affect observed cases and incidence rates though with a delay in observed cases.

To control for confounding effects arising from climate conditions and spatial dependence from workplace mobility, we include average temperature ($Temperature_{i,t}$) and workplace mobility ($Mobility_{i,t}$) by municipality and week. Both variables enter with a one week lag (where *L.* denotes the lag operator), to allow pathogens to develop before PCR tests identify infections. Finally, deterministic linear and non-linear (quadratic) time trends specific to region types ($RegType_{r(i)}$) control for divergent urban-rural infection dynamics specific for social and economic structures that are not attributable to the treatment. The region type indicator ($RegType_{r(i)}$) classifies municipalities into *R*=4 categories (urban, intermediate urban, rural and periphery) based on regional demographics like population density and social structure. Finally, in equation ($A$.1), $\tau_t$ denote week-fixed effects common to all municipalities (to cover cyclical trends in human SARS-CoV-2 infections in Denmark), and $\mu_i$ are municipality-fixed effects controlling for unobservable regional heterogeneity that may confound outcomes beyond the level of region types (e.g. to cover differences in infection levels in regions with external borders); $\varepsilon_{i,t}$ is the error term.

***Alternative treatment indicators.*** We focus on estimating the coefficient $\delta_1$ which captures the effect of mink farm infections on subsequent human SARS-CoV-2 incidence rates. This treatment indicator $(InfFarm_{i,t})$ is constructed in three different ways to assess the robustness of the obtained results. By default, it is a binary absorbing indicator specified as $InfFarm_{i,t} = \mathbb{1}[t \geq InfFarm_i^{first}]$, i.e. the dummy takes values of one from calendar week *t* onwards when the first SARS-CoV-2 mink farm infection $(InfFarm_i^{first})$ was reported in municipality *i* and stays at this value for treated municipalities during the remainder sample period. This is our default specification.

One may conjure that the two-way infection dynamics between animals and humans associated with a disease outbreak on a mink farm phases out over time and that treatment effects are thus only transitory in nature. As an alternative specification we therefore define the binary treatment indicator $(InfFarm_{i,t})$ in a non-absorbing way, i.e. that it reverts back to zero for the affected municipality *n* weeks after the first SARS-CoV-2 mink farm infection $(InfFarm_i^{first})$. A new infection on another farm in municipality *i* during this

$n$-week period extend the treatment period subsequently. We perform sensitivity analyses by considering alternatives as $n$=3 or $n$=4 weeks, which appear reasonable if treatment effects are assumed to be static with an incubation time of approx. 1-2 weeks and an equally lengthened infection duration. We also check the sensitivity of the empirical results with regard to alternative data sources: While our default absorbing treatment indicator is defined on the basis on published data obtained from the home page of the Danish Veterinary and Food Administration, we also use updated process data from the same administration to define treatment indicators on a rolling basis. Process data has the advantage that it accounts for near-time adjustments in the reporting of SARS-CoV-2 infections on mink farms and culling of mink, while it may come at the cost of being preliminary. Taken together, we believe that utilizing all available data from the Danish Veterinary and Food Administration offers the best way to arrive at robust results.

Finally, rather than using a binary flag indicator, a third continuous specification of $InfFarm_{i,t}$ counts the stock of SARS-CoV-2 infected farms in municipality $i$ by accumulating newly reported mink farm infections in each municipality until week $t$. In analogy to the binary treatment indicator, we either specify a non-reverting stock by summing over all infected farms and weeks. Alternative, we accumulate infections over $n$=3 or $n$=4 weeks. Different ways to calculate cumulative stocks are presented in Figure A2.

***Panel event study (PES).*** A panel event study is applied as a complementary fully flexible estimation approach to allow treatment effects to vary by week rather than treatment having an absorbing or non-absorbing static structure with underlying assumptions as in the DiD approach.[3-6] By considering time-heterogeneity in the estimated coefficient of the treatment indicator $(InfFarm_{i,t})$ we are able to identify dynamic treatment effects. It may mirror, for instance, a time delay in the effect from two-way infection dynamics between mink and humans through transmission from e.g. persons living or working at mink farms on the wider human population in a municipality and the gradual implementation non-pharmaceutical interventions in terms of culling mink and a local lockdown. It may also account for accumulating effects over time. The PES is estimated as

$$Y_{i,t} = \sum_{j=-N}^{M} \delta_j \, IF_{i,t}^{j} + \ \beta_1 \cdot Cum\_Infectious_{i,t} + \ \beta_2 \cdot WCum\_Infectious_{i,t} + \ \tau \cdot Cum\_Tested_{i,t}$$

$$+ \vartheta \cdot L.Temperature_{i,t} \ + \ \lambda \cdot L.Mobility_{i,t} + \sum_{r=1}^{R} \gamma_r \cdot \left(RegType_{r(i)} \times Trend_t\right)$$

$$+ \sum_{r=1}^{R} \gamma_r \cdot \left(RegType_{r(i)} \times Trend_t\right) \ + \sum_{r=1}^{R} \alpha_r \cdot \left(RegType_{r(i)} \times Trend_t^2\right) \ + \tau_t + \mu_i + \varepsilon_{i,t}, \qquad (A.2)$$

where $IF_{i,t}^{j}$ is a set of binary weekly treatment indicators defined relative to the treatment start in municipality $i$, i.e. the occurrence of the first SARS-CoV-2 mink farm infection $\left(InfFarm_i^{first}\right)$, with

$$IF_{i,t}^{j} = \begin{cases} \mathbb{1}\left[t \leq InfFarm_i^{first} + j\right] & \text{if} \quad j = -N \\ \mathbb{1}\left[t = InfFarm_i^{first} + j\right] & \text{if} \quad -N < j < M \\ \mathbb{1}\left[t \geq InfFarm_i^{first} + j\right] & \text{if} \quad j = M. \end{cases}$$

The index $j = -N, \ldots, M$ denotes the maximum number of periods before first treatment (-*N*) and after first treatment (*M*) in municipality *i*. The inclusion of several periods before first treatment is observed allows us to test for early anticipation effects in the outcome variable prior to first treatment. If such effects are significant and positive, a rise in the incidence rate in municipality *i* is likely driven by other latent factors rather than our treatment in focus. In the absence of early anticipation effects, however, significant effects arriving with the treatment start in the included *M* periods after the first treatment, can be taken as evidence for an effect of disease outbreaks on mink farms on the epidemiological trend in affected municipalities. Plotting the weekly treatment effects ($\delta_j$) allows us to time the phasing-in of this transmission channel and identify potential intervening effects associated with the local lockdown and culling of mink. Estimated effects beyond this time interval are accumulated in a single coefficient shown in the first pre- and last post-treatment period, which is typically referred to as binning.[6] The treatment indicator $IF_{i,t}^{j}$ for the last pre-treatment observation ($j = -1$) is omitted to capture the baseline difference between treated and non-treated municipalities. Moreover, as an extension to the above specification we also include neighbouring regions in the treatment group to identify not only direct but also indirect treatment effects.

## References

1 Lechner M. The Estimation of Causal Effects by Difference-in-Difference Methods. Found. Trends Econ. 2010; 4: 165–224.

2 Wing C, Simon K, Bello-Gomez RA. Designing Difference in Difference Studies: Best Practices for Public Health Policy Research. Annu. Rev. Public Health 2018; 39: 453-469.

3 Athay S, Imbens G. Design-based Analysis in Difference-In-Differences Settings with Staggered Adoption. 2018, Working Paper 24963, National Bureau of Economic Research. https://www.nber.org/papers/w24963.

4 Borusyak K, Jaravel X. Revisiting Event Study Designs. 2020, Working Paper, Harvard University. https://scholar.harvard.edu/borusyak/publications/revisiting-event-study-designs.

5 Goodman-Bacon A. Difference-in-Differences with Variation in Treatment Timing. 2018, Working Paper 25018, National Bureau of Economic Research. https://www.nber.org/papers/w25018.

6 Schmidheiny K, Siegloch S. On Event Study Designs and Distributed-Lag Models: Equivalence, Generalization and Practical Implications. 2019, CESifo Working Paper Series 7481, CESifo.

## B. Descriptive statistics and additional estimation results

**Table A1: Summary statistics for treatment indicators related to SARS-CoV-2 outbreaks on mink farms and non-pharmaceutical policy interventions (culling of mink and local lockdowns)**

| | **Non-treated municipalities** | | **Treated municipalities (with SARS-CoV-2 outbreaks on mink farms)** | |
|---|---|---|---|---|
| **Variable** | **Mean** | **Standard Deviation** | **Mean** | **Standard Deviation** |
| Number of mink farms per municipality | 2.46 | 3.73 | 38.54 | 25.97 |
| Number of mink farms with SARS-CoV-2 outbreaks per week by municipality | 0 | 0 | 0.43 | 1.53 |
| Cumulative number of mink farms with SARS-CoV-2 outbreaks per week by municipality (permanent) | 0 | 0 | 3.89 | 8.99 |
| Cumulative number of mink farms with SARS-CoV-2 outbreaks per week by municipality (3 weeks rolling basis) | 0 | 0 | 1.29 | 3.69 |
| Cumulative number of mink farms with SARS-CoV-2 outbreaks per week by municipality (4 weeks rolling basis) | 0 | 0 | 1.71 | 4.59 |
| Number of mink livestock on SARS-CoV-2 infected mink farms per week by municipality | 0 | 0 | 6,472.99 | 21,907.49 |
| Cumulative number of mink livestock on SARS-CoV-2 infected mink farms per week by municipality (permanent) | 0 | 0 | 56.73 | 120.08 |
| Cumulative number of mink livestock on SARS-CoV-2 infected mink farms per week by municipality (3 weeks rolling basis) | 0 | 0 | 19.42 | 51.58 |
| Cumulative number of mink livestock on SARS-CoV-2 infected mink farms per week by municipality (4 weeks rolling basis) | 0 | 0 | 25.78 | 63.86 |
| Number of SARS-CoV-2 infected farms subject to culling of mink per week by municipality | 0 | 0 | 0.43 | 2.14 |
| Number of culled livestock of mink on SARS-CoV-2 infected farms per week by municipality | 0 | 0 | 6,472.99 | 30,451.27 |
| Lockdown (share of municipalities affected by lockdown; 1=100%) | 0 | 0 | 0.07 | 0.26 |

*Notes:* Source information for variables are given in the main manuscript.

**Table A2: Summary statistics for outcomes (human SARS-CoV-2 incidence and positivity rates)**

| Variable | Non-treated municipalities | | Treated municipalities (with SARS-CoV-2 outbreaks on mink farms) | |
|---|---|---|---|---|
| | Mean | Standard Deviation | Mean | Standard Deviation |
| Number of human SARS-CoV-2 cases in municipality; (weekly) | 47.12 | 181.59 | 26.30 | 48.76 |
| Municipal population level (total persons) | 60,148.15 | 80,847.26 | 57,154.83 | 43,713.38 |
| Human incidence rate (SARS-CoV-2 cases per 100,000 population; weekly) | 68.26 | 109.41 | 44.56 | 60.48 |
| Positivity rate (% share of human SARS-CoV-2 infections in all tested persons; weekly) | 0.93 | 1.04 | 0.72 | 0.82 |

*Notes:* Source information for variables are reported in the main manuscript.

**Table A3: Summary statistics for regional control variables used for DiD and PES estimations**

| Variable | Non-treated municipalities | | Treated municipalities (with SARS-CoV-2 outbreaks on mink farms) | |
|---|---|---|---|---|
| | Mean | Standard Deviation | Mean | Standard Deviation |
| Human SARS-CoV-2 infections by municipality (stock over last two weeks) | 102.25 | 372.34 | 64.44 | 114.74 |
| Number of PCR tests per week by municipality | 8095.62 | 16287.95 | 7625.47 | 8864.01 |
| Workplace mobility (index; relative to Jan-Feb. 2020; weekly by municipality) | -20.82 | 11.51 | -18.01 | 11.51 |
| Average temperature (in degree Celsius; weekly by municipality) | 9.69 | 3.63 | 9.60 | 3.47 |
| Share of urban regions (Type 1, 1=100%) | 0.46 | 0.50 | 0.04 | 0.20 |
| Share of intermediate urban regions (Type 2, 1=100%) | 0.20 | 0.40 | 0.08 | 0.28 |
| Share of rural regions (Type 3, 1=100%) | 0.26 | 0.44 | 0.50 | 0.50 |
| Share of peripheral regions (Type 4, 1=100%) | 0.08 | 0.27 | 0.38 | 0.48 |

*Notes:* Source information for variables are given in the main manuscript.

**Figure A1: Infected mink farms and culling of mink farms**

Panel A: Newly reported SARS-CoV-2 outbreaks on mink farms by municipality, weeks 24-51

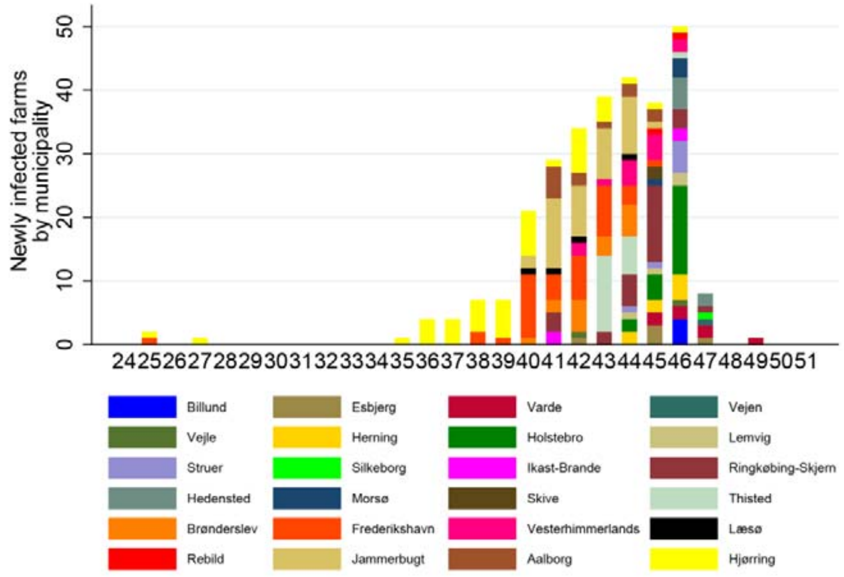


Panel B: Number of mink farms subject to culling by municipality, weeks 24-51

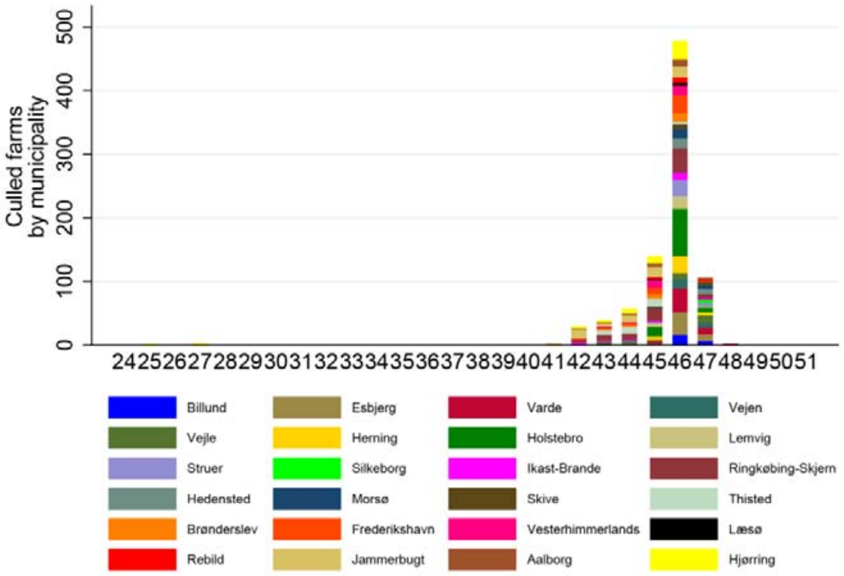


*Source:* Fødevarestyrelsen, Smittede mink farme uge for uge (in Danish), Smittede minkfarme uge for uge (foedevarestyrelsen.dk) and process data from Fødevarestyrelsen on culling on mink farms. Retrieved on April 8 2021.

**Figure A2: Different presentations of cumulative stocks of infected mink farms by municipality**

Panel A: Cumulative stocks of infected mink farms by municipality and week defined on permanent basis

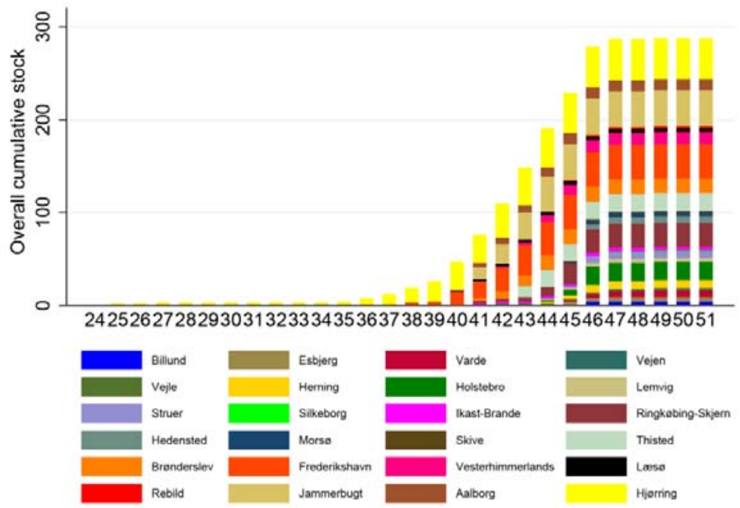


Panel B: Cumulative stocks of infected mink farms by municipality and week defined on rolling basis

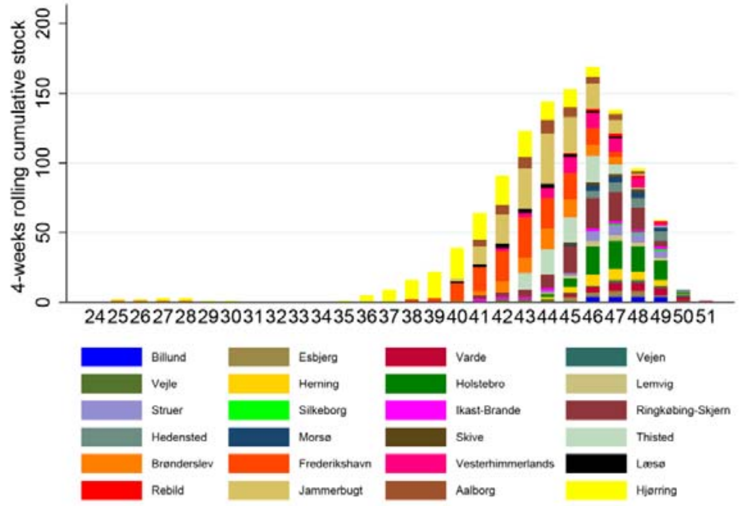


*Notes:* The permanent cumulative stock of infected farms adds the number of infected farms by week to the total cumulative sum for each municipality. The cumulative stock calculated on a rolling basis shown in Panel B accumulates the number of infected farms over the last four weeks building on the assumption of temporally diminishing effects from a given SARS-CoV-2 outbreak on a mink farm in the light of public health interventions. Alternative specifications use a three week period; results can be obtained upon request from the authors or generated through the replication files linked to this paper.

*Source:* Fødevarestyrelsen, Smittede mink farme uge for uge (in Danish), Smittede minkfarme uge for uge (foedevarestyrelsen.dk), Retrieved April 8 2021.

**Table A4: Difference-in-Difference estimation results for SARS-CoV-2 positivity rate**

| **Sample:** | **(1)** **All municipalities** | **(2)** **All municipalities** | **(3)** **All municipalities** | **(4)** **Only municipalities with mink farms** |
|---|---|---|---|---|
| Infected farms (absorbing binary) | 0.65 [0.358,0.946] | | | 0.41 [0.0860,0.7429] |
| Infected farms* (non-absorbing, binary) | | 0.58 [0.339,0.817] | | |
| Infected farms* (continuous) | | | 0.05 [0.032,0.069] | |
| Neighbour (50 km) Infected farms | 0.32 [0.078,0.556] | 0.45 [-0.025,0.925] | 0.03 [-0.029,0.086] | 0.10 [-0.345,0.545] |
| Lockdown Northern Jutland (binary) | -0.87 [-1.187,-0.557] | -0.88 [-1.167,-0.591] | -0.76 [-1.022,-0.496] | -1.01 [-1.324,-0.686] |
| Neighbour (50 km) Lockdown Northern Jutland | -0.14 [-0.399,0.130] | -0.14 [-0.391,0.114] | -0.03 [-0.305,0.238] | -0.72 [-1.492,0.052] |
| Culling on infected mink farms (binary) | -0.37 [-0.694,-0.042] | -0.22 [-0.457,0.016] | 0.003 [-0.178,0.186] | -0.21 [-0.564,0.136] |
| Neighbour (50km) Culling on infected mink farms | -0.20 [-0.445,0.048] | -0.05 [-0.235,0.131] | 0.10 [-0.063,0.254] | -0.02 [-0.240,0.192] |
| Stock of infectious human population (continuous) | 0.000 [0.0001,0.0003] | 0.000 [0.0001,0.0002] | 0.000 [0.0001,0.0002] | 0.000 [0.0000,0.0007] |
| Neighbour (50km) Stock of infectious human population | 0.002 [0.0012,0.0026] | 0.001 [0.0007,0.0022] | 0.002 [0.0010,0.0024] | 0.001 [0.0002,0.0016] |
| Workplace mobility (continuous) | 0.003 [-0.0024,0.0092] | 0.005 [-0.0009,0.0106] | 0.004 [-0.0017,0.0094] | 0.003 [-0.0046,0.0114] |
| Average temperature (continuous) | -0.04 [-0.092,0.015] | -0.01 [-0.063,0.042] | -0.02 [-0.068,0.038] | -0.07 [-0.128,-0.006] |
| No. of observations | 2632 | 2632 | 2632 | 1680 |
| No. of clusters (municipalities) | 94 | 94 | 94 | 60 |
| Within $R^2$ | 0.767 | 0.771 | 0.771 | 0.711 |
| Linear and quadratic time trends by region types | Yes | Yes | Yes | Yes |
| Week and municipal fixed effects | Yes | Yes | Yes | Yes |

*Notes:* 95% confidence intervals in brackets. * = variables defined on a three week rolling basis; neighbour values for the variable "Infected farms" are measured in the same dimension as the underlying variable in each column.

**Table A5: Difference-in-Difference estimation results for alternative treatment indicators (full sample)**

| Outcome: Incidence rate<br>Sample: | (1)<br>All municipalities | (2)<br>All municipalities | (3)<br>All municipalities | (4)<br>All municipalities |
|---|---|---|---|---|
| Infected farms[#]<br>(non-absorbing binary) | 48.23<br>[23.809,72.651] | | | |
| Infected farms[#]<br>(continuous) | | 4.47<br>[2.651,6.282] | | |
| Infected farms[$]<br>(continuous) | | | 1.41<br>[-0.208,3.024] | |
| Infected mink livestock[#]<br>(continuous) | | | | 0.36<br>[0.225,0.486] |
| Neighbour (50 km)<br>Infected farms | 19.27<br>[-16.896,55.426] | 4.08<br>[-0.973,9.128] | 0.03<br>[-6.155,6.217] | 0.33<br>[0.013,0.640] |
| Lockdown Northern<br>Jutland (binary) | -47.91<br>[-82.312,-13.499] | -42.24<br>[-74.431,-10.057] | -70.56<br>[-1.2e+02,-18.797] | -41.10<br>[-72.703,-9.487] |
| Neighbour (50 km)<br>Lockdown Northern Jutland | -23.84<br>[-54.252,6.574] | -11.29<br>[-39.704,17.123] | -17.59<br>[-45.984,10.801] | -9.16<br>[-38.386,20.060] |
| Culling on infected<br>mink farms (binary) | -16.32<br>[-41.585,8.946] | 2.52<br>[-14.732,19.765] | 15.95<br>[-1.813,33.713] | -3.31<br>[-21.335,14.707] |
| Neighbour (50km)<br>Culling on infected mink farms | -2.80<br>[-19.808,14.214] | 6.19<br>[-9.913,22.292] | 8.73<br>[-8.184,25.650] | 3.90<br>[-12.138,19.939] |
| Stock of infectious human<br>population (continuous) | 0.07<br>[0.027,0.110] | 0.06<br>[0.027,0.094] | 0.07<br>[0.028,0.114] | 0.06<br>[0.027,0.092] |
| Neighbour (50km)<br>Stock of infectious human<br>population | 0.16<br>[0.075,0.246] | 0.17<br>[0.091,0.252] | 0.20<br>[0.121,0.282] | 0.17<br>[0.086,0.248] |
| Cumulative number of PCR<br>Tests (continuous) | -0.001<br>[-0.0022,-0.0004] | -0.001<br>[-0.0016,-0.0003] | -0.001<br>[-0.0022,-0.0004] | -0.001<br>[-0.0016,-0.0003] |
| Workplace mobility<br>(continuous) | 0.40<br>[0.016,0.774] | 0.37<br>[-0.013,0.748] | 0.31<br>[-0.087,0.703] | 0.37<br>[-0.008,0.753] |
| Average temperature<br>(continuous) | -7.86<br>[-13.056,-2.668] | -7.45<br>[-12.709,-2.186] | -9.05<br>[-14.314,-3.784] | -7.36<br>[-12.667,-2.050] |
| No. of observations | 2632 | 2632 | 2632 | 2632 |
| No. of clusters<br>(municipalities) | 94 | 94 | 94 | 94 |
| Within $R^2$ | 0.778 | 0.781 | 0.775 | 0.775 |
| Linear and quadratic time<br>trends by region types | Yes | Yes | Yes | Yes |
| Week and municipal fixed<br>effects | Yes | Yes | Yes | Yes |

*Notes:* 95% confidence intervals in brackets. # = variables defined on a four weeks rolling basis; $ = variables defined on a permanent basis; neighbour values for the variable "Infected farms" are measured in the same dimension as the underlying variable in each column.

**Table A6: Difference-in-Difference estimation results for alternative treatment indicators (subsample)**

| **Outcome: Incidence rate**<br>**Sample:** | **(1)**<br>**Only municipalities with mink farms** | **(2)**<br>**Only municipalities with mink farms** | **(3)**<br>**Only municipalities with mink farms** | **(4)**<br>**Only municipalities with mink farms** | **(5)**<br>**Only municipalities with mink farms** |
|---|---|---|---|---|---|
| Infected farms*<br>(non-absorbing binary) | 55.02<br>[29.944,80.086] | | | | |
| Infected farms#<br>(non-absorbing binary) | | 43.37<br>[18.940,67.797] | | | |
| Infected farms#<br>(continuous) | | | 3.83<br>[2.053,5.601] | | |
| Infected farms$<br>(continuous) | | | | 1.14<br>[-0.323,2.610] | |
| Infected mink livestock#<br>(continuous) | | | | | 0.31<br>[0.178,0.435] |
| Neighbour (50 km)<br>Infected farms | 34.94<br>[-2.080,71.961] | 21.87<br>[-13.080,56.821] | 2.84<br>[-2.126,7.799] | -0.09<br>[-5.411,5.231] | 0.19<br>[-0.135,0.519] |
| Lockdown Northern<br>Jutland (binary) | -47.44<br>[-80.863,-14.009] | -49.39<br>[-83.368,-15.402] | -48.20<br>[-77.983,-18.422] | -69.45<br>[-1.2e+02,-19.663] | -47.49<br>[-75.691,-19.289] |
| Neighbour (50 km)<br>Lockdown Northern Jutland | -72.74<br>[-1.5e+02,7.261] | -80.15<br>[-1.6e+02,0.642] | -47.88<br>[-1.3e+02,33.556] | -72.41<br>[-1.6e+02,11.887] | -40.58<br>[-1.2e+02,41.752] |
| Culling on infected<br>mink farms (binary) | -15.66<br>[-39.684,8.363] | -12.47<br>[-36.791,11.842] | 4.72<br>[-9.934,19.364] | 16.27<br>[0.523,32.012] | -0.48<br>[-15.855,14.892] |
| Neighbour (50km)<br>Culling on infected mink farms | -10.79<br>[-25.530,3.957] | -8.30<br>[-23.718,7.115] | 2.41<br>[-11.125,15.947] | 2.81<br>[-11.692,17.306] | 1.27<br>[-12.550,15.095] |
| Stock of infectious human<br>population (continuous) | 0.12<br>[0.064,0.171] | 0.13<br>[0.066,0.185] | 0.11<br>[0.059,0.160] | 0.14<br>[0.068,0.203] | 0.11<br>[0.059,0.154] |
| Neighbour (50km)<br>Stock of infectious human population | 0.02<br>[-0.054,0.095] | 0.02<br>[-0.053,0.100] | 0.02<br>[-0.042,0.091] | 0.06<br>[-0.012,0.122] | 0.02<br>[-0.047,0.087] |
| Cumulative number of PCR<br>Tests (continuous) | -0.002<br>[-0.0026,-0.0008] | -0.002<br>[-0.0029,-0.0009] | -0.001<br>[-0.0022,-0.0006] | -0.002<br>[-0.0029,-0.0010] | -0.001<br>[-0.0021,-0.0006] |
| Workplace mobility<br>(continuous) | 0.06<br>[-0.368,0.497] | 0.01<br>[-0.435,0.456] | -0.08<br>[-0.486,0.326] | -0.17<br>[-0.597,0.265] | -0.07<br>[-0.476,0.335] |
| Average temperature<br>(continuous) | -4.34<br>[-8.313,-0.358] | -5.60<br>[-9.523,-1.680] | -5.07<br>[-9.062,-1.084] | -7.27<br>[-11.273,-3.273] | -4.95<br>[-8.922,-0.985] |
| No. of observations | 1680 | 1680 | 1680 | 1680 | 1680 |
| No. of clusters (municipalities) | 60 | 60 | 60 | 60 | 60 |
| Within $R^2$ | 0.751 | 0.744 | 0.750 | 0.738 | 0.753 |
| Linear and quadratic time trends by region types | Yes | Yes | Yes | Yes | Yes |
| Week and municipal fixed effects | Yes | Yes | Yes | Yes | Yes |

*Notes:* 95% confidence intervals in brackets. * = variables defined on a three week rolling basis; # = variables defined on a four weeks rolling basis; $ = variables defined on a permanent basis; neighbour values for the variable "Infected farms" are measured in the same dimension as the underlying variable in each column.

**Figure A3: Robustness tests for dynamic treatment effects based on alternative treatment indicator**

Panel A: Municipalities within top-5 percentile for number of infected mink farms (direct)

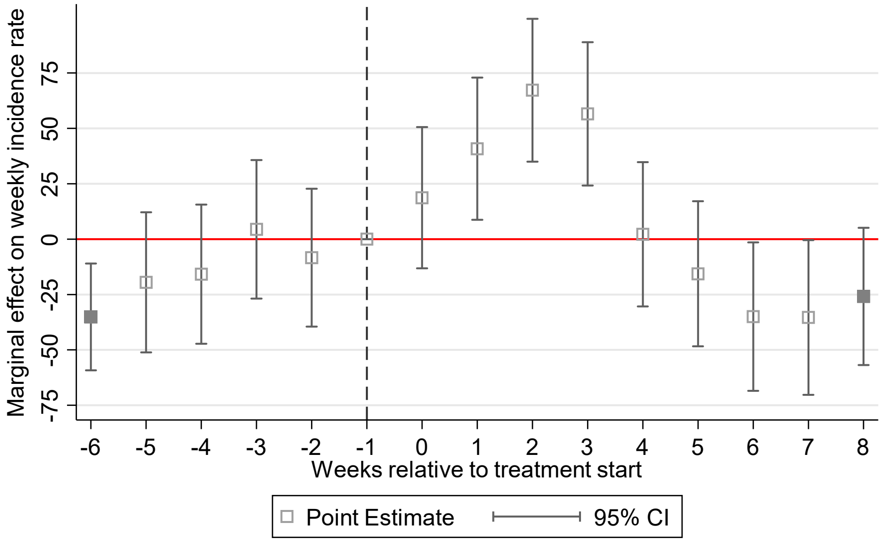


Panel B: Municipalities within top-5 percentile for number of infected mink farms (direct + spatial)

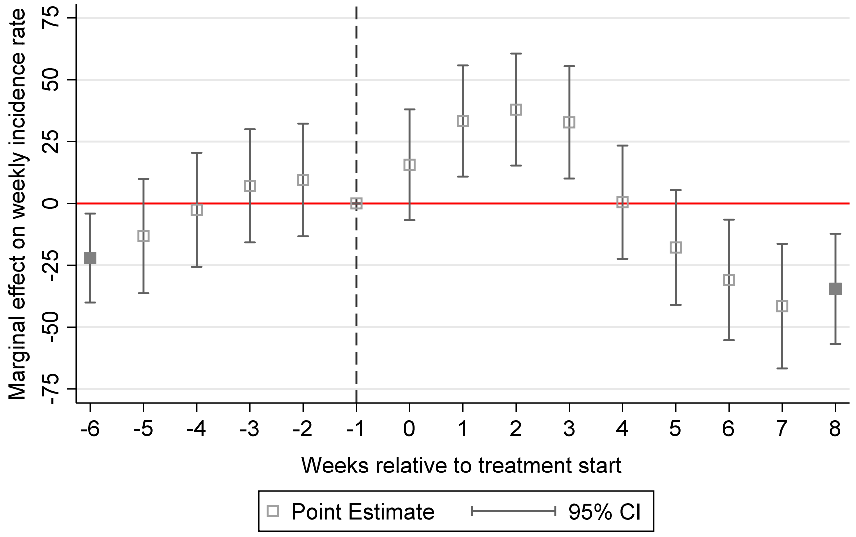


*Notes:* CI = Confidence Interval; dotted vertical lines indicate the last pre-treatment observation (baseline); dynamic treatment effects obtained from panel event study (PES) controlling for the stock of infectious individuals in municipality and spatial neighbourhood; number of PCR tested persons, (lagged) temperature, workplace mobility, region-type specific trends and region- and time-fixed effects. The last pre-treatment observation is omitted to capture the baseline difference between treated and non-treated municipalities. Treated municipalities are restricted to those belonging to the top-5 percentile of SARS-CoV-2 outbreaks on mink farms per municipality.

## C. Background on public health interventions related to SARS-CoV-2 outbreaks on Danish mink farms

**Box A1: Timeline of major interventions to suppress the risk of human infections from mink**

**Mid-June 2020**: First mink farms infected in the Danish region of Northern Jutland in north-western Denmark; culling of mink at infected farms.

**July 7, 2020**: Mink strategy #1: No culling of mink on infected farms. Monitoring of infected farms and introducing biosecurity precautions in terms of face masks, gloves, hand disinfection and change of cloth among workers at farms.

**October 1, 2020**: Mink strategy #2: Culling of mink at infected farms. Mink at 100 farms in Northern Jutland are to be culled, as 41 are infected.

**November 4, 2020**: CULLING: Due to an overall assessment of infection risk from lessons learned all mink in Denmark is to be gradually culled. LOCKDOWN: Seven severely affected municipalities in Northern Jutland subject to: 1) government encourages absence of travel between municipalities and beyond, 2) public employed working from home, 3) primary school from 5$^{th}$ to 8$^{th}$ grade send home, 4) public transport closed down, 5) private employers encouraged to let employees work from home unless in manufacturing, 6) intensified testing and tracking, 7) cultural and leisure activities closed down.

**November 16, 2020**: Lockdown of seven municipalities is eased as of November 19 in terms of mobility between seven municipalities but not beyond. Primary school from 5$^{th}$ to 8$^{th}$ grade reopened as of November 23. All mink in Northern Jutland have been culled - both infected and non-infected.
